# Mapping the Endogenous Ketogenic System Across Ages, Sex and Diets

**DOI:** 10.1093/geroni/igab046.3506

**Published:** 2021-12-17

**Authors:** Brenda Eap, Mitsunori Nomura, Oishika Panda, Thelma Garcia, John Newman

**Affiliations:** 1 Buck Institute for Research on Aging, Buck Institute for Research on Aging, California, United States; 2 Buck Institute for Research on Aging, Buck Institute of Research on Aging, California, United States; 3 Buck Institute for Research on Aging, Novato, California, United States

## Abstract

Understanding how our cells maintain energy homeostasis has long been a focus of aging biology. A decline in energy metabolism is central to many age-related diseases such as Alzheimer’s disease, heart failure, frailty, and delirium. Intervening on pathways involved in energy homeostasis can extend healthy lifespan. When our primary energy substrate glucose, is scarce, our bodies use ketone bodies (i.e. beta-hydroxybutyrate, acetoacetate, acetone). Aging is associated with glucose intolerance and insulin insensitivity, yet what role ketone body metabolism might play in compensating for impaired glucose utilization in age-related diseases is understudied. Here, we investigated how the body’s endogenous ketone body production and utilization pathways are modulated by age across the lifespan of female and male C57BL/6 mice (4 mo old, 12 mo old, 22 mo old). We show how different ages have different metabolic and gene expression responses to 1-week ketogenic diet (KD) or ketone ester diet. We observed an apparently compensatory ketogenic response in older animals fed normal diet, with a stronger compensatory response driven by KD. We observed tissue-specific changes, including induction of ketone body production enzymes in the aging heart. When comparing the ketogenic capacity between sexes, females had a higher basal level and less variation with age, underscoring the importance of sexual dimorphism in metabolism. Overall, these findings suggest that older animals use ketone bodies to meet energetic demands in a normal diet context. This study supports the potential roles of ketogenic therapies such as exogenous ketones to improve energy homeostasis in conditions of aging.

